# Structure and Properties of Self-Reinforced Polytetrafluoroethylene-Based Materials

**DOI:** 10.3390/polym17121609

**Published:** 2025-06-09

**Authors:** Shunqi Mei, Oksana Ayurova, Undrakh Mishigdorzhiyn, Vasily Kornopoltsev, Evgeny Kovtunets, Kirill Demin, Bair Garmaev, Andrei Khagleev

**Affiliations:** 1 Hubei Digital Textile Equipment Key Laboratory, Wuhan Textile University, Wuhan 430073, China; mishigdorzh@gmail.com; 2 Department of Inorganic and Organic Chemistry, Banzarov Buryat State University, 670000 Ulan-Ude, Russia; 3 Institute of Physical Materials Science, Siberian Branch of the Russian Academy of Sciences, 670047 Ulan-Ude, Russia; kirill.demin.19992@gmail.com (K.D.); bair.garmaev@gmail.com (B.G.); khagleev@yandex.ru (A.K.); 4 Baikal Institute of Nature Management, Siberian Branch of the Russian Academy of Sciences, Sakhyanovoy Str. 6, 670047 Ulan-Ude, Russia; kompo@mail.ru (V.K.); kovtunets@gmail.com (E.K.)

**Keywords:** polytetrafluoroethylene, self-reinforced polymer composites, polymer waste

## Abstract

A promising direction in polymer material processing is the development of self-reinforced polymer composites (SRPMs), representing a relatively new group of composite materials. The self-reinforcement method allows for materials of one polymer to be combined with different molecular, supramolecular, and structural features. The high adhesive and mechanical properties of SRPMs are due to the formation of a homogeneous system with no inter-phase boundary. Moreover, self-reinforcement considers the possibility of using polymer waste to create high-strength composites, which reduces the environmental load. In the current work, the phase composition, structure, and properties of SRPMs based on polytetrafluoroethylene (PTFE) were studied. SRPMs were prepared by mixing industrial and regenerated PTFE powders and then subjected to pressing and sintering. Two types of regenerated PTFE were used for the SRPM preparation: a commercial PTFE of the TOMFLON^TM^ trademark and mechanically grinded PTFE waste. The degree of crystallinity of the obtained materials (41–68%) was calculated by XRD analysis; the crystallite size was determined to be 30–69 nm. Thermal analysis of the composites was carried out by the DSC method in the temperature range of 25–370 °C. The characteristics of thermal processes in self-reinforced composites correlate with the data from structural studies of XRD and FTIR analyses. The results of dynamic mechanical analysis showed that the introduction of regenerated PTFE powder into an industrial one increased the elasticity modulus from 0.6 GPa up to 2.0–3.1 GPa. It was shown that the phase state of the SRPMs depended on the method of processing polymer waste (the type of regenerated PTFE) that determined the heat resistance and mechanical properties of the obtained composite material.

## 1. Introduction

The use of recycled materials as a new resource base is one of the most dynamically developing areas of polymer materials processing [1,2]. Self-reinforcement is a promising method of material processing that enables the manufacture of high-strength thermoplastic polymer composites [3,4]. Self-reinforced polymer composites (SRPMs) offer numerous advantages, including thermoformability, high strength and rigidity, excellent impact resistance at low density, and high biodegradability [3,5,6]. For example, studies have shown that SRPMs based on polylactic acid (PLA) exhibit improved mechanical properties and biodegradability, making them promising for eco-friendly applications [7]. A distinctive feature of this type of composite is their possibility of being completely processed, since they are made from a single polymer material that serves both as a matrix and a reinforcing element, which allows for the recycling of used products from self-reinforced composites without the need for separation, as in other types of composite materials [8,9,10]. Due to the relative uniformity in this single-component system, an ideal interaction between the matrix and the reinforcing component is achieved, facilitating stress transfer between the matrix and the filler and providing better adhesion compared to heterogeneous composites [3]. Chemical modification of the fiber or matrix surface can further enhance these properties by strengthening the interfacial interaction [11]. A combination of different crystalline forms of amorphous crystalline polymers (polymorphism), different supramolecular structures, or different grades of the same polymer can be used to obtain SRPMs. For example, a monopolymer composite may contain a more rigid phase as a reinforcing element and a less rigid phase as a matrix. Modern production methods, such as hot pressing with controlled fiber alignment, allow for the optimization of the structure of these phases to achieve maximum strength [12]. Kmetty et al. [4] present the classification of SRPMs in accordance with their components (single- or multicomponent), their production (single-stage or multi-stage procedures), and the spatial alignment of the reinforcing phase in the matrix (in one, two, or three dimensions). Additional studies refine the impact of these spatial configurations on mechanical properties, emphasizing the importance of fiber orientation [13]. Gao et al. [14] focus on developing polymer–polymer composites, including biomaterials and composites from renewable resources, design principles and mechanisms, as well as preparation methods and applications. For example, SRPMs find application in the automotive industry due to their lightweight and high-strength properties, which contribute to reducing vehicle weight and improving energy efficiency [15]. Various methods for design and production have been researched and developed, including hot impact, overheating, dissolution, partial dissolution, cold drawing, physical processing, and chemical modification. Polymer waste processing products can also be considered for producing self-reinforced materials, depending on the required properties of the composite material, highlighting their versatility and potential for a closed-loop production cycle [3,16,17].

It is known that PTFE and its slightly modified derivatives account for about 60–65% of the total global fluoropolymer market, while its production increases by about 7% annually, due to its unique properties: excellent thermal stability, chemical inertia, exceptionally low coefficient of friction, and good dielectric properties [18,19]. Recently, work has been progressing successfully to create new forms of PTFE that are free from the disadvantages of the base polymer, in particular, its low wear resistance [19,20]. The structure and properties of the modified forms, and hence the possibilities and areas of their application, largely depend on their production technology. Therefore, there is a need for a thorough study of each product obtained in a new way. The difference in the morphology of fluoropolymer particles is due to the products obtained by different processing methods having varying ratios of molecular components, each designed to form specific morphological structures, leading to differences in the physical and mechanical properties [19].

The rheological properties of PTFE limit the use of traditional technologies for producing precision parts. It is generally accepted that the melting point of the polymer is 327 °C. However, at this temperature, its viscosity is very high (about 10^10^–10^12^ Pa s) [8], so PTFE does not pass into a viscous flow state even at the decomposition temperature (415 °C). This means that when heated, it does not spread and retains its shape. The high viscosity of the PTFE melt prevents the use of processing methods such as extrusion and injection molding. Therefore, the main processing method is the mechanical processing of blanks. During this process, waste is generated in the form of chips and scrap, resulting in environmental and economic problems. In this regard, the task of recycling polymer waste into marketable products or raw materials for reuse becomes a demanding issue [3,17,21]. The few studies in the field of self-reinforced materials using regenerated polymers primarily focus on changes in deformation and strength properties, depending on the processing cycles [3,17]. There are no works studying the influence of polymer waste processing methods on the chemical and physical–mechanical properties of self-reinforced materials.

In this regard, this article examines the influence of PTFE waste recycling methods on the structure of SRPMs and changes in thermal, deformation-strength, and tribological properties.

## 2. Materials and Methods

**Figure 1:**
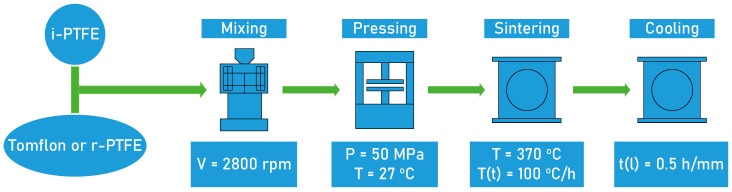
Scheme for obtaining self-reinforced materials.

SRPMs were prepared by mixing industrial powders and regenerated PTFE in a high-speed paddle mill at a speed of 2800 rpm (Figure 1). The content of regenerated PTFE in the polymer composite was 5, 10, 20, and 30 wt.%. The polymer mixture was formed by cold pressing (27 °C, 50 MPa) followed by free sintering at 370 ± 5 °C in an air furnace (heating rate: 100 °C/h, exposure: 0.5 h per 1 mm thickness of the sample, cooling in a closed oven).

Industrial PTFE (further i-PTFE) produced by TD Kirovo-Chepetsk Chemical Company Ltd., Kirovo-Chepetsk, Russia [22]. The degree of crystallinity calculated using the TOPAS 4.2 software package was 54.0% [21]. The heating thermogram of the i-PTFE showed a single endotherm with a maximum at 335.6 °C and an enthalpy of −23.8 J/g; the cooling thermogram showed an exotherm with a maximum at 308.4 °C and an enthalpy of 21.02 J/g.

Two types of recycled PTFE were used to prepare the SRPMs. The first was a powder of regenerated (or recycled) PTFE (further r-PTFE) obtained by the mechanical grinding of polymer waste by abrasion on a corundum stripping wheel at a linear sliding speed of 27 m/s and at a load of 1 MPa in the designed installation mentioned in [23]. The degree of crystallinity calculated using the TOPAS 4.2 software package was 66.5% [21]. Melting of the regenerated polymer powder occurred at 339.6 °C with an enthalpy of −60.92 J/g, and crystallization at 305.7 °C with an enthalpy of 50.47 J/g.

The second type of recycled PTFE was an ultrafine PTFE of the TOMFLON^TM^ trademark (further Tomflon), which is a loose, crumbly white powder with a particle size of ~5 µm [24], manufactured by Fluoropolymer Technologies LLC (Tomsk, Russia). It was obtained through a combination method of recycling PTFE waste, which involved both radiation and mechanical treatment. Accelerated electrons performed radiation treatment and led to the accumulation of defects, which initiated the appearance of micro- and macro-cracks in the polymer. During the subsequent mechanical processing of the material in jet mills, these defects caused the particles to break apart. As a result, particles in the form of ribbons were formed, which were fully consistent in molecular structure with the structure of industrial samples of PTFE [21]. The degree of crystallinity calculated using the TOPAS 4.2 software package was 81.0% [21]. The melting point of Tomflon was 335.3 °C, and the melting enthalpy was −49.5 J/g; polymer crystallization took place at a temperature of 305.3 °C with an enthalpy of 49.62 J/g.

The particle sizes of the r-PTFE were determined on a SALD-7500nano nanometer (Shimadzu, Tokyo, Japan). Morphological studies were performed using a scanning electron microscope (SEM) JSM-6000 (JEOL Ltd., Tokyo, Japan). The XRD data of the samples were obtained at room temperature on a D2 PHASER (Bruker AXS GmbH, Karlsruhe, Germany) diffractometer at CuKa radiation, a shooting interval of 2θ = 4–70°, and a scanning step—0.02°. The processing of experimental data by full-profile analysis methods, calculating the degree of crystallinity and the size of crystallites, was performed using the TOPAS 4.2 software package. The Double-Voigt approach implemented in TOPAS 4.2 also enabled the calculation of the average strain (γ) based on the contributions of the size and strain of individual crystallites to the line profile shapes as a function of 2θ, accounting for Lorentzian and Gaussian line broadening.

IR measurements were performed on an FT-805 IR Fourier spectrometer (SIMEX Scientific and Production Company LLC, Novosibirsk, Russia) in the absorption range of 4000–550 cm^−1^ using specular–diffuse reflection in the scientific and technological laboratory of Roknlab LLC (Ulan-Ude, Russia). The IR spectra were obtained by the Kramers–Kronig transformation.

The thermal characteristics of the initial components and self-reinforced composites based on them were determined using a synchronous thermal analyzer STA 449 F1 Jupiter (Netzsch, Selb, Germany) at the Center for Collective Use of the Baikal Institute of Nature Management, the Siberian Branch of the Russian Academy of Sciences. The measurements were carried out in platinum crucibles in the heating–cooling mode up to 370 °C at a heating rate of 10 °C/min. The flow rates of the main gas used were 50 mL/min of Ar and 20 mL/min of protective Ar. The sample weights ranged from 17 to 23 mg. The areas of their peaks determined the heat of melting and crystallization.

The thermophysical properties (accumulation modulus *E*′, loss modulus *E*″, tangent of the mechanical loss angle tg *δ*) of the samples were determined on a dynamic mechanical analyzer DMA 242 C (Netzsch, Selb, Germany) in the temperature range of 25–500 °C at a heating rate of 5 K·min^−1^ in the penetration mode; the diameter of the penetrating end of the punch was 3 mm. The samples for thermophysical testing were obtained by pressing under a pressure of 50 MPa and by the subsequent sintering of the samples at T = 370 ± 5 °C in a furnace in an air atmosphere using standard technology (heating rate of 100 deg h^−1^, holding for 0.5 h per 1 mm of sample thickness, cooling in a closed furnace, normalization at room temperature for 1 day) in the form of tablets with a diameter of 10 mm and a thickness of 5 mm.

The deformation and strength properties were determined according to [25] on an INSTRON 3367 (Illinois Tool Works Inc., Frankfort, IL, USA) universal machine at room temperature and a moving speed of 200 mm/min. The samples for the tensile test were obtained by pressing according to the aforementioned modes in the form of standard blades (type 2). The number of samples per test was 5.

The wear test used the block-on-ring scheme with dry sliding friction on the SMC-2 friction machine (Tochpribor LCC, Moscow, Russia). The rotation speed of the counterbody was 0.8 m/s and the load on the friction pair was 200 N. A hardened disk made of high-speed steel R6M5 (analog to AISI M2 steel) with a hardness of 60 ± 2 HRC was used as the ring. The relative wear rate of the samples was determined as the weight loss of the sample per unit time (mg/h) due to abrasion. The weight loss of the samples was measured on an AXIS AGN-200 analytical balance (Gdańsk, Poland). The measurement error was ±0.002 g. The samples for tribological testing were obtained by pressing according to the aforementioned modes in the form of rectangles measuring 20 × 12 × 10 mm^3^.

## 3. Results

**Figure 2:**
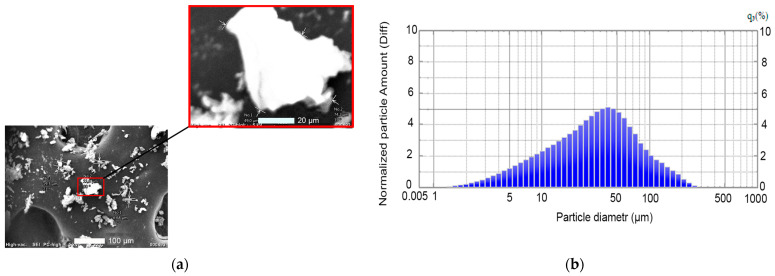
A microimage (**a**) and a diagram of the crushed polytetrafluoroethylene waste powder (**b**).

Figure 2 shows a microimage of the ground PTFE waste powder. The particle sizes of the PTFE powder produced by mechanical abrasion vary widely and range from 0.5 to 250 µm. According to the diagram, the average particle size is 17 µm (Figure 2b).

**Figure 3:**
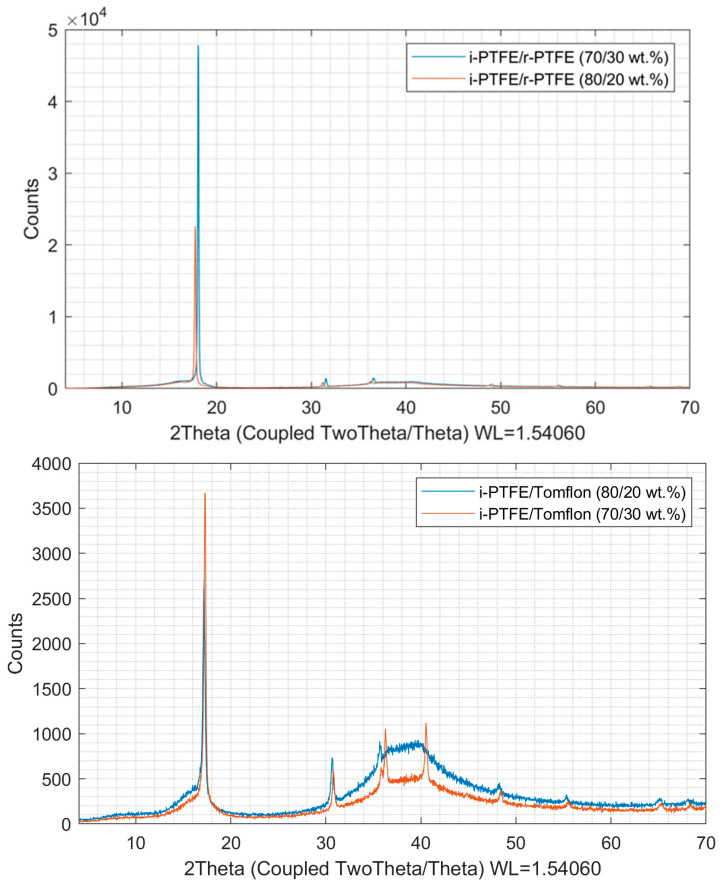
Diffraction patterns of self-reinforced materials.

The XRD method determined the degree of crystallinity of the obtained SRPMs (Figure 3). The results of the profile analysis of the studied samples are presented in Table 1.

Based on the data obtained, the quantitative ratios of the crystalline and amorphous phases of the SRPMs with the compositions i-PTFE/r-PTFE and i-PTFE/Tomflon were calculated, as well as the degree of crystallinity depending on the technology used for processing the initial components (Table 1). Many studies report a change in the degree of crystallinity of polymers depending on the processing methods [26,27,28,29]. However, there are no data on the crystallinity of polymer mixtures using regenerated polymers.

To simulate the effects of the microstructure based on X-ray data, TOPAS 4.2 software was used. The size of the crystallites was estimated using Formula (1), independent of the shape of the crystallites proposed by Stokes and Wilson [30], using integral half-widths *β_i_*:

(1) $$\beta_{i}=\lambda /L_{Vol}\cos\theta$$

where *λ* is the wavelength, *θ* is the Bragg angle, *β_i_* is the integral half-width of the line profile adjusted by the device, and *L_Vol_* is the volume-weighted average column height (crystallite size). This concept leads directly to a formula identical to the Scherrer equation, except that the constant *k* takes the value of one.

The calculated crystallite size values for the obtained self-reinforced materials are shown in Table 1. It can be seen that the size of the crystallites (*L_Vol_*) of the obtained SRPMs depends on the method of processing PTFE waste. Thus, self-reinforced composites obtained using Teflon were characterized by a higher degree of crystallinity and a smaller crystallite size. This may be because the Tomflon production method, which combines radiation and mechanical treatment, forms short polymer chains that crosslink to form small crystallites [27].

At the same time, the results of XRD show that the degree of crystallinity of the obtained self-reinforced materials depends on the type of regenerated polymer and increases with its content. The crystallinity of SAM based on Tomflon, characterized by a higher degree of crystallinity (81%), is higher in comparison with SAM based on r-PTFE with a degree of crystallinity (66.5%). In a complex interaction, factors such as chain length and overall molecular structure coordinate to determine the degree of movement available to the polymer chain. The more mobile the polymer chain, the easier it is for the polymer to transition from an amorphous to a crystalline state. Earlier in our work [14], we showed that the initial fluoropolymers obtained using different processing methods are characterized by a three-phase structure (a crystalline phase and two amorphous components) and have different ratios of molecular components. It is assumed that the ratio of amorphous phases in the initial fluoropolymers contributes to the formation of certain morphological formations in the self-reinforced material. Thus, for r-PTFE, an increased content of amorphous phase I (32.3%) is observed in comparison to amorphous phase II (3.7%), while for Tomflon, on the contrary, amorphous phase I contains 10.1%, and amorphous phase II—46.9%. Amorphous phase I is the “normal” amorphous phase of the polymer, consisting of entangled chains of macromolecules, and phase II is a low-molecular-weight amorphous formation with a molecular structure different from PTFE chains. The increase in crystallinity for i-PTFE/Tomflon compared to i-PTFE/r-PTFE may be attributed to the enhanced molecular mobility of short Tomflon chains, which facilitates the improved packing of polymer chains.

**Figure 4:**
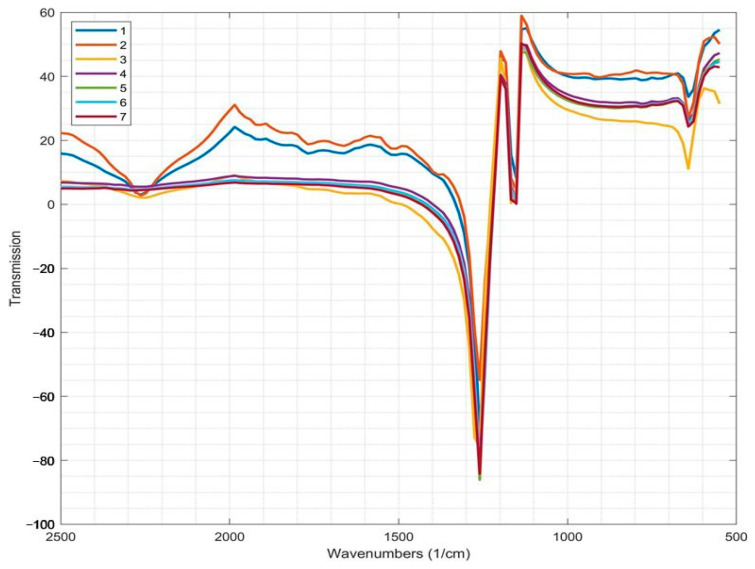
The IR spectra of the samples: 1—i-PTFE; 2—r-PTFE; 3—Tomflon; 4—i-PTFE/r-PTFE (80/20 wt %); 5—i-PTFE/Tomflon (80/20 wt %); 6—i-PTFE/r-PTFE (70/30 wt %); 7—i-PTFE/Tomflon (70/30 wt %).

Figure 4 shows the IR spectra of industrial PTFE, PTFE waste processed in various ways, and self-reinforced materials based on them. Intense absorption bands related to valence vibrations of -CF_2_- (1165 cm^−1^) and -CC- (~1265 cm^−1^) groups are observed in the IR spectrum of industrial PTFE (Figure 4). In the region of wave numbers below 650 cm^−1^, deformation and extraplanar fluctuations of the -CF_2_- groups are observed: fan oscillations of *γω* (-CF_2_-) are manifested at 639 cm^−1^ and deformation vibrations at 555 cm^−1^. The absorption bands in the range of 2300–1350 cm^−1^ correspond to fluctuations in the end -CF=CF_2_ groups and groups of branches characteristic of low-molecular-weight fractions of the polymer. A well-defined doublet is observed in the PTFE spectrum at 640 and 625 cm^−1^ (bands of order, bands of crystallinity). The observed effect is associated with a change in the spiral conformation of macromolecules and their packaging. The band at 625 cm^−1^ characterizes the defect structure, i.e., the chain sections where transitions between left- and right-rotating spirals occur, and the band at 640 cm^−1^ reflects the presence of a regular spiral in the polymer structure. The molecular conformations of the PTFE chain include 136 spirals, 157 spirals, 157 disordered spirals, etc.; i.e., 157 spirals means that 15 units of -CF_2_- are present in 7 chain turns. Thermal activation led to structural transformations of the PTFE chain (from 136 spirals to 157 spirals) and occurs when the temperature rises to 19 °C. The low-intensity absorption bands observed at 782 and 721 cm^−1^ refer to vibrations of molecular chains in the amorphous phase of the polymer. The absorption band at 782 cm^−1^ is also used to determine the degree of order in the PTFE structure. The assignment of bands in the IR spectra was carried out following known data [19,27,31,32]. The IR spectra of recycled PTFE waste (r-PTFE and Tomflon) (Figure 4) show typical absorption bands of the polymer at 1165 and 1265 cm^−1^, corresponding to valence vibrations of the -CF_2_- and -CC- groups, and at 639 and 555 cm^−1^, associated with fan and deformation vibrations of the -CF_2_- groups. There is no noticeable difference between the IR spectra of PTFE waste recycled differently. Thus, the regenerated PTFE has a similar chemical structure regardless of the method used for its processing. However, some papers [19,27] report that certain transformations leading to changes in polymer properties occur at the molecular level. It has been shown that polytetrafluoroethylene’s morphology and supramolecular structure change after treatment while maintaining the chemical structure of the polymer [19,27]. As expected, the IR spectra of recycled PTFE waste (r-PTFE and Tomflon) show a change in the intensity of the absorption bands (Figure 4). Some differences are observed when comparing the IR spectra of industrial and regenerated PTFE (r-PTFE and Tomflon), which consist of increasing the intensity of the absorption bands at 640 and 555 cm^−1^. Thus, for regenerated fluoropolymers (r-PTFE and Tomflon) characterized by a high degree of crystallinity (66.5 and 81%, respectively), there is an increase in the intensity of the crystallinity band (640 cm^−1^) and its shift to the short-wavelength region of the spectrum. The study showed that the optical density of the band at 782 cm^−1^, observed in the IR spectra of the samples, increases linearly with the decreasing degree of crystallinity of the sample. Similar results were obtained in [13,17]. The absorption band *ν* (-CC-) at 1265 cm^−1^ in the infrared spectrum of PTFE is shifted to the HF region in the infrared spectrum of Teflon (1271 cm^−1^). In the case of self-reinforced polymer composites, there is a change not only in the intensity of the absorption bands but also in the shape of the IR spectra. Some additional absorption bands in the range of 2300–1350 cm^−1^, related to fluctuations of the end groups and branch groups, are absent in the IR spectra of self-reinforced materials. The observed expansion of the absorption band at 1165 cm^−1^ is probably due to the formation of many dispersed crystallites in the polymer structure, which do not dominantly affect the degree of crystallinity but form a structure that most effectively resists sample destruction and, consequently, wear (20–66%). A similar effect was shown in [13]. As a result of mechanical action, the polymer transitions to dispersed, nanodisperse and other structural states. IR spectroscopic studies of all samples showed the preservation of the chemical structure characteristics of PTFE. The processing methods used to produce r-PTFE and Tomflon lead to a slight break in the macromolecular chain; however, in some cases, certain changes occur at the molecular level, which leads to an increase in the physical and mechanical properties of the polymer.

**Figure 5:**
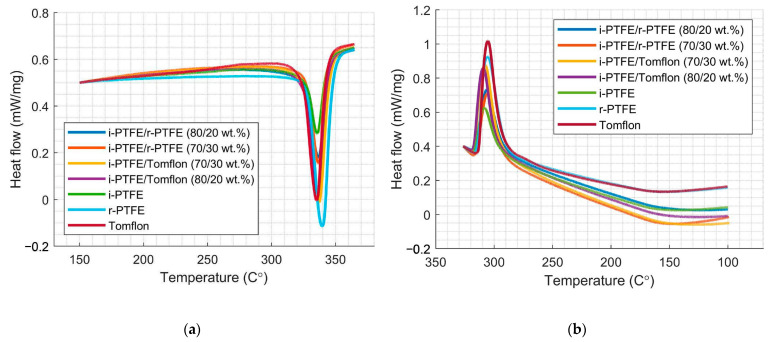
Thermograms of DSC showing the melting peak (**a**) and crystallization peak (**b**) of raw and self-reinforced materials.

The results of the differential scanning calorimetry (DSC) data in Figure 5 show the endothermic effects of melting and the exothermic effects of crystallization for samples of initial fluoropolymers and SRPMs based on them. The thermal effects show a clear change in SRPMs compared to the original components.

The enthalpy of crystallization corresponds to the energy released during the formation of intermolecular bonds in the crystalline phase of the polymer [33]. It can be seen that for the material with the composition i-PTFE/Tomflon (70/30 wt %), characterized by a higher degree of crystallinity (68.6%), the highest heat of crystallization is observed, which is also due to the formation of many small crystalline formations (Table 2). An increase in the enthalpy of crystallization is associated with both an increase in the degree of crystallinity and the formation of intermolecular bonds at the crystallite–crystallite interfaces, which contribute to the energy released during the formation of the crystalline phase. The melting enthalpy is the energy required to break the intermolecular bonds in the crystal lattice of a polymer. The decrease in melting enthalpy is most likely due to the destruction of the crystal structure. In several self-reinforced composites obtained, the melting enthalpy decreases as the degree of crystallinity decreases. One of the reasons for the decrease in melting temperature may be the formation of small polymer crystallites in materials. However, the resulting material of the composition i-PTFE/Tomflon (70/30 wt %), characterized by small crystallites, has a higher melting point. However, it is shown (Table 2) that this material is characterized by a higher microstress (*γ*) occurring in crystallites. A study [20] suggested that a melting peak at a higher temperature may be associated with the melting of crystals that are disoriented when heated. The DSC analysis data are consistent with the results of XRD structural studies and FTIR analysis.

Relaxation processes in polymers are of significant interest for gaining a better understanding of the long-term stability of potential products made from these materials. Relaxation processes can be initiated by various external influences, including temperature, mechanical stress, hydrostatic pressure, and electric and magnetic fields, i.e., they are implemented in almost all processes of production, processing, and the application of polymeric materials [34,35].

**Figure 6:**
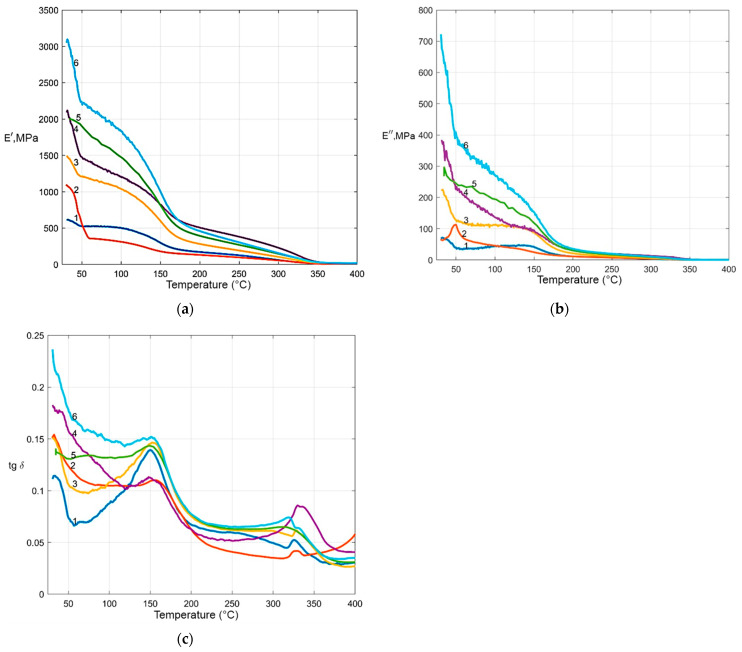
Thermophysical properties: (**a**) is the modulus of elasticity; (**b**) is the loss modulus; (**c**) is the tangent of the angle of mechanical losses. SRPMs of the composition: 1—i-PTFE; 2—r-PTFE; 3—Tomflon; 4—i-PTFE/Tomflon (80/20 wt %); 5—i-PTFE/r-PTFE (80/20 wt %); 6—i-PTFE/Tomflon (70/30 wt %).

The greatest dynamic losses are observed in the temperature ranges of both solid-phase (~25–40 °C) and melting (~335–340 °C)-phase transitions, as well as relaxation (~150 °C) transitions (Figure 6). In the region of ~25–40 °C, *β*-relaxation is observed, associated with phase transformations occurring in the crystalline region of the polymer. The type I phase transition at 25–30 °C is caused by changes in the crystallite’s unit cell parameters. The type II solid-phase transition at ~40 °C is associated with the loss of the helical chirality of the long-chain polytetrafluoroethylene crystal molecule, i.e., with the loss of the symmetry element of the crystal [36,37]. A study [31] reported that at atmospheric pressure and a temperature of several tens of degrees, PTFE is in three solid-phase states (II, IV, and I). In phase II, at temperatures below 19 °C, the polymer has a well-ordered triclinic unit cell. Macromolecules have a spiral conformation of 13/6 (units per turn). At 19 °C, an “order–disorder” transition occurs, which is a reorientation (rotation) of macromolecules around their axes. The molecules are slightly unwound and adopt the conformation 15/7. This intermediate phase (IV) has a metric-hexagonal unit cell and persists up to 30 °C. Above 30 °C, further rotational disordering and unwinding of the spirals occur as the temperature increases. In this phase I, the spiral conformation 15/7 gradually gives way to the averaged conformation 2/1 (flat zigzag). Figure 6b shows that the intensity of the β-relaxation peaks of polymer composites increases with increasing crystallinity. Similar results are shown in [36].

For the obtained SRPMs, with an increase in the degree of crystallinity, stiffness (modulus of elasticity *E*^/^) increases by 2–4 times compared to PTFE (Figure 6a). This can be explained by the fact that crystallites with a new morphology are formed [37]. The difference in the morphology of SRPMs is due to the initial fluoropolymers obtained through different processing methods, which have varying ratios of molecular components, each designed to form specific morphological formations [19]. In current research, it has been shown that an SRPM with the composition i-PTFE/Tomflon (70/30 wt %), characterized by high crystallinity and the smallest size of crystallites, has a significant modulus of elasticity of 3.1 GPa. In study [38], the authors demonstrated that the modulus of elasticity increases with the concentration of crystallites, and the smaller the crystallite size, the greater the modulus of elasticity of the polymer. The maximum hardening is observed in the region above the relaxation transition temperature of polytetrafluoroethylene of ~25 °C. The increased elasticity of the material (*E*′) at 25–34 °C is accompanied by maximum losses of mechanical energy (*E*″) due to its dissipation in the form of heat (Figure 6b). As the temperature increases, there is a gradual decrease in the modulus of elasticity associated with the transition of the material from a glassy state to a highly elastic one. At ~150 °C, the amorphous phase of the material is glassy (α-relaxation), which is expressed in the appearance of a peak [39,40]. As the degree of crystallinity of polymer composites increases, the α-peak expands and becomes asymmetric (Figure 6b). This indicates that an increase in the degree of crystallinity in the sample leads to significant restrictions on the mobility of polymer chain segments in the amorphous region of the material, which determines this relaxation process [40]. In the amorphous phase, intense molecular movements begin at temperatures above the glass transition temperature, while in the crystalline phase, these movements are much weaker. Significant changes in the properties of composites are observed in the region of the first-order phase transition. At a melting temperature of 335–340 °C, the crystalline phase of the material disappears, and the elasticity of the sample decreases sharply (Figure 6a,b). 

**Figure 7:**
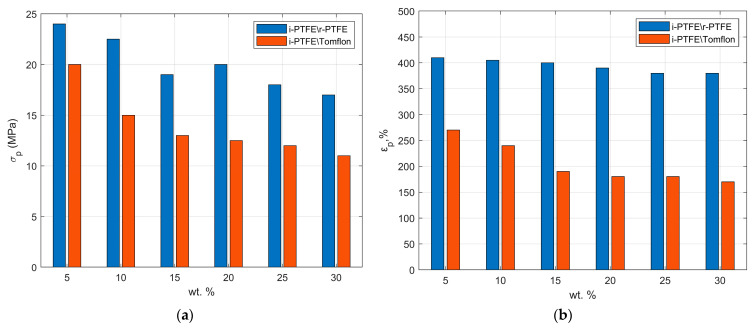
Tensile strength (*σ_p_*) (**a**) and elongation at break (*ε_p_*) (**b**) of SRPMs.

The effect of the self-reinforcement of industrial PTFE with regenerated polymer on mechanical properties is considered. It is established that the deformation and strength properties of the obtained self-reinforced composites for the entire range of compositions differ from the deformation and strength properties of industrial PTFE (Figure 7), decreasing with the increasing content of regenerated powder. So, at a 5-% SRPM composition (i-PTFE/r-PTFE), the tensile strength and elongation are at the level of the properties of industrial PTFE (*σ_r_* = 23.5 MPa; *ε_p_* = 418%), whereas when using Tomflon powder (even with its small introduction), there is a sharp decrease in deformation and strength properties in the two. When r-PTFE powder is introduced, mechanical properties deteriorate to a lesser extent than Tomflon powder, which is associated with polymer waste processing technology. With the mechanical crushing of PTFE waste by simple abrasion, partial mechanical destruction of the polymer is possible, which is accompanied by a slight decrease in molecular weight. In the radiation method for producing Tomflon powder, polymer destruction occurs due to the rupture of macromolecules under the action of a stream of electrons or gamma quanta, which leads to a decrease in molecular weight by ~10–30 times, and, as a result, to a decrease in the deformation and strength properties [21]. Study [36] describes the stress–strain behavior for a semi-crystalline polymer as a physical reflection of the competition of two deformation mechanisms: initial cold drawing with the rotation of crystalline domains inside the amorphous polymer phase, maximizing the tilt and sliding of the crystalline chain, and then orientation hardening, observed as deformation hardening when the available rotation and sliding of crystalline domains are decreasing. Similar deformation behavior is shown for PTFE. Amorphous regions are oriented when stretched, and sliding occurs along parallel grooves in crystalline regions. When sliding occurs, the crystal regions are oriented by rotating the long axis along the direction of the load. At higher deformations, the crystals bend or bend around the grooves, which leads to orientation hardening.

The stretching of the obtained self-reinforced composites occurs at the initial deformation stage due to amorphous regions of the polymer. In the crystalline regions, the partial destruction of crystallites and the movement of the resulting smaller structural fragments in the direction of deformation occur due to the straightening of sections of the through chains. Deformation occurs due to the movement of crystallites along slip planes and defects in the direction of the load until the sample is destroyed. Despite the relatively high crystallinity, low strength values are observed for the obtained materials. At the last stage of deformation, corresponding to the deformation of the oriented crystalline polymer, the destruction significantly depends on the molecular weight of the polymer. Thus, the decrease in material strength is due to the rupture of polymer chains with a low molecular weight in the regenerated polymer. The contribution of each of the processes considered to the general mechanism of deformation of a crystalline polymer depends on the structure of the polymer, the ratio of amorphous and crystalline components, and the nature of the crystalline structures present in it.

**Figure 8:**
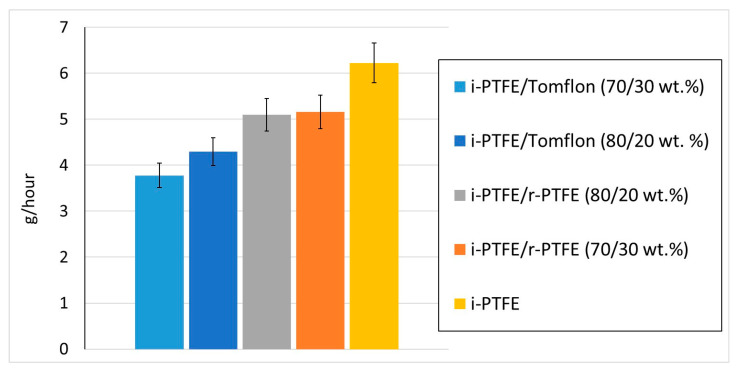
The intensity of the mass wear of SRPMs.

Several studies focus on changing PTFE’s physical and chemical properties to increase wear resistance using crosslinking by gamma irradiation [27,28,29,41,42], which correlates with a decrease in wear. At the same time, this method can decrease the chemical stability, tensile strength, and toughness of PTFE [42]. It is reported that crystallinity plays an important role in the tribological behavior of polymers. It has been shown that an increase in crystallinity reduces the friction force and the depth of irregularities [20]. The results of tribo-tests show that the wear resistance of the obtained self-reinforced materials increases by 20–66% compared to industrial PTFE (Figure 8). The best wear resistance is observed for self-reinforced i-PTFE/Tomflon materials. It is possible that small wear particles form during the wear process, forming thinner portable films, which contributes to the improvement of tribological characteristics [43,44].

It was shown in [43] that the most effective fillers for reducing wear in PTFE are weak aggregates that can decompose into nanoscale components at the sliding boundary, limiting the wear product size. Therefore, it is assumed that a sample of the i-PTFE/Tomflon composition with a large number of microstresses in crystallites (0.327) easily dissociates into smaller fragments during sliding, forming a thin, portable film, thereby improving the wear resistance of the composite [44].

## 4. Conclusions

Mixing industrial PTFE with its waste products is a practical way to obtain SRPMs with applicable complex properties. The initial fluoropolymers obtained by different processing methods have different ratios of molecular components, and each contributes to the construction of certain morphological formations in the self-reinforced material. A comparative study of composites has shown that the thermophysical and mechanical properties depend on the processing method and the content of the regenerated polymer. With an increase in the degree of crystallinity, an increase by 66–78% in the modulus of elasticity of materials is observed and in wear resistance by 20–66% relative to industrial polytetrafluoroethylene, possibly due to the formation of small crystallites. The results of the deformation and strength characteristics show the possibility of using r-PTFE obtained by mechanical abrasion in the filling range from 5 to 30 wt % to create SRPMs with good performance properties (*σ_p_* = 17–24 MPa; *ε_p_* =370–410%). The deformation and strength properties of industrial PTFE, self-reinforced with Tomflon powder, decrease by almost 2 times due to the low molecular weight of the regenerated polymer.

## Figures and Tables

**Table 1:** Phase composition of the obtained polymer materials.

Sample	Phase Domain	Area
i-PTFE/r-PTFE (80/20 wt %)	Amorphous phase I (17.55°2θ)	6148.2595
	Amorphous phase II (38.62°2θ)	11,151.8073
	The crystalline phase	4410.188
Degree of crystallinity * (%) = 41.8; crystallite size (*L_Vol_*, nm) = 69.22		
i-PTFE/Tomflon (80/20 wt %)	Amorphous phase I (18.04°2θ)	8256.87617
	Amorphous phase II (38.76°2θ)	15,611.7727
	The crystalline phase	8788.76292
Degree of crystallinity (%) = 57.7; crystallite size (*L_Vol_*, nm) = 38.419		
i-PTFE/r-PTFE (70/30 wt %)	Amorphous phase I (16.27°2θ)	1601.54357
	Amorphous phase II (37.48°2θ)	9018.54478
	The crystalline phase	2185.21767
Degree of crystallinity (%) = 51.6; crystallite size (*L_Vol_*, nm) = 74.632		
i-PTFE/Tomflon (70/30 wt %)	Amorphous phase I (9.8°2θ)	2215.12779
	Amorphous phase II (37.13°2θ)	7116.64923
	The crystalline phase	4841.37617
Degree of crystallinity (%) = 68.6; crystallite size (*L_Vol_*, nm) = 30.09		

* The degree of crystallinity was calculated as the ratio of the fraction of the crystalline phase to the fraction of the crystalline and amorphous phase I.

**Table 2:** Structural and thermal characteristics of the obtained SRPMs.

SRPMs	The Size of Crystals, *L_Vol_* nm	Microstresses, *γ*	*T_melt_*, °C	∆*H_melt_*, J/g	*T_cr_*, °C	∆*H_cr_*, J/g
i-PTFE/ r-PTFE (80/20 wt %)	69.22	0.181	336.6	−40.55	306.1	29.36
i-PTFE/ r-PTFE (70/30 wt %)	74.63	0.114	336.8	−40.71	305.7	30.88
i-PTFE/Tomflon (80/20 wt %)	38.42	0.182	335.6	−46.46	309.4	34.47
i-PTFE/Tomflon (70/30 wt %)	30.09	0.327	337.0	−60.97	307.9	40.58

## Data Availability

Data are contained within the article.
